# Proinflammatory-activated glioma cells induce a switch in microglial polarization and activation status, from a predominant M2b phenotype to a mixture of M1 and M2a/B polarized cells

**DOI:** 10.1042/AN20130045

**Published:** 2014-05-08

**Authors:** Lucia Lisi, Egidio Stigliano, Libero Lauriola, Pierluigi Navarra, Cinzia Dello Russo

**Affiliations:** *Institute of Pharmacology, Catholic University Medical School, Rome, Italy; †Institute of Pathology, Catholic University Medical School, Rome, Italy

**Keywords:** arginase, glioma, IL-10, microglia, microglial polarization, mTOR, NOS, ARG, arginase, BBB, blood–brain barrier, BrdU, bromodeoxyuridine or 5-bromo-2-deoxyuridine, CM, conditioned media, C-CM, control-conditioned media, CNS, central nervous system, COX, cyclooxygenase, DMEM, Dulbecco’s modified Eagle’s medium, GBM, glioblastoma multiforme, H_2_DCF-DA, 2′,7-dichlorodihydrofluorescein diacetate, IFN, interferon, IL, interleukine, iNOS, inducible nitric oxide synthase, LI-CM, LPS–IFNγ-conditioned media, LPS, lipopolysaccharide, mTOR, mammalian target of rapamycin, MTS, 3-(4,5-dimethylthiazol-2-yl)-5-(3-carboxymethoxyphenyl)-2-(4-sulfophenyl)-2*H*-tetrazolium, TGF, transforming growth factor, TNF, tumor necrosis factor

## Abstract

Malignant gliomas are primary brain tumors characterized by morphological and genetic complexities, as well as diffuse infiltration into normal brain parenchyma. Within gliomas, microglia/macrophages represent the largest tumor-infiltrating cell population, contributing by at least one-third to the total tumor mass. Bi-directional interactions between glioma cells and microglia may therefore play an important role on tumor growth and biology. In the present study, we have characterized the influence of glioma-soluble factors on microglial function, comparing the effects of media harvested under basal conditions with those of media obtained after inducing a pro-inflammatory activation state in glioma cells. We found that microglial cells undergo a different pattern of activation depending on the stimulus; in the presence of activated glioma-derived factors, i.e. a condition mimicking the late stage of pathology, microglia presents as a mixture of polarization phenotypes (M1 and M2a/b), with up-regulation of iNOS (inducible nitric oxide synthase), ARG (arginase) and IL (interleukine)-10. At variance, microglia exposed to basal glioma-derived factors, i.e. a condition resembling the early stage of pathology, shows a more specific pattern of activation, with increased M2b polarization status and up-regulation of IL-10 only. As far as viability and cell proliferation are concerned, both LI-CM [LPS (lipopolysaccharide)–IFNγ (interferon γ) conditioned media] and C-CM (control-conditioned media) induce similar effects on microglial morphology. Finally, in human glioma tissue obtained from surgical resection of patients with IV grade glioblastoma, we detected a significant amount of CD68 positive cells, which is a marker of macrophage/microglial phagocytic activity, suggesting that *in vitro* findings presented here might have a relevance in the human pathology as well.

## Abstract 2

We have characterized the influence of glioma-soluble factors on microglial, comparing the effects of media harvested under-basal conditions to those of media obtained after inducing a pro-inflammatory activation in glioma cells. Our data suggest that microglia might exert different effects on glioma depending on the stage of disease.

## INTRODUCTION

Primary brain tumors represent approximately 2% of all adult neoplasia; of these, most common phenotype is the high-grade malignant glioma (grade III–IV by the World Health Organization) (Wen and Kesari, 2008). Malignant gliomas are primary CNS (central nervous system) tumors, originating from glial cells and characterized by morphological and genetic complexity as well as diffuse infiltration of tumor cells into the normal brain parenchyma (Rolle et al., 2012). Current therapeutic strategies (i.e., aggressive surgical resection combined with radiation and chemotherapy) are largely ineffective; patients have a life expectancy of 14 months from the initial diagnosis of GBM (glioblastoma multiforme), the most aggressive glioma type of tumor (Stupp et al., 2005, 2009). In fact, malignant gliomas remain at present among the most lethal cancers (for a recent review Bradley and Rees, 2014), and this underscores the need for novel and more effective therapeutic approaches.

It has been widely shown that gliomas are histologically heterogeneous tumors, containing cancerous cells mostly derived from astrocytes and oligodendrocytes, but also stromal cells and a significant amount of different inflammatory cells; the latter have been found to significantly contribute to glioma cell growth and progression (Charles et al., 2012). Among tumor-infiltrating cells, microglia/macrophages represent the largest population of cells, contributing to at least one-third of the total tumor mass (Carvalho da Fonseca and Badie, 2013). The percentage of microglia within the tumor positively correlates with the tumor grade, with the highest proportion (78%) found in proliferative and high-grade glioma (Roggendorf et al., 1996). Furthermore, the abundance of microglia is also positively related to cancer invasiveness (Markovic et al., 2005). In the last decade, a large number of experimental studies provided evidence for a tumor-supporting role of microglia; on the opposite, data in support of microglial anti-tumor properties have also been reported (Li and Graeber, 2012). Interestingly, the group of Seyfried hypothesized that some neoplastic cells within the GBM arise from the transformed macrophage/microglia (Huysentruyt et al, 2011), further suggesting the tumor-supporting hypothesis of microglia. However, the exact role of microglia in the biology of malignant gliomas remain to be fully elucidated (Zhai et al., 2011).

Under pathological conditions microglia do not constitute a uniform cell population, but rather comprise a family of cells with diverse phenotypes, which may be associated to beneficial or, on the contrary, to detrimental biological activities (Schwartz et al., 2006). Microglial-activated cells can be broadly divided into classically activated M1 cells, with cytotoxic properties and alternatively activated M2 cells, with phagocytic activities. Furthermore, the M2 activation can be further divided into three classes: M2a, involved in repair and regeneration; M2b, an immune-regulatory phenotype; or M2c, an acquired-deactivating phenotype (Chhor et al., 2013). Each of these distinct microglia phenotypes has specific inducers: Toll-like receptor-4 agonist LPS (lipopolysaccharide), IFNγ (interferon γ) and TNFα (tumor necrosis factor α) for M1; IL (interleukine)-4 and IL-13 for M2a; immune complexes and toll-like receptor agonists, as well as IL-1R ligands for M2b; IL-10, TGFβ (transforming growth factor β) and glucocorticoids for M2c (for a recent review, see Boche et al., 2013). Several reports have shown that intra-tumoral microglia express an M2 phenotype in response to soluble factors released by glioma cells. For example, Kaminska and co-workers have recently characterized the M2 activation state of rat microglia cells exposed to a conditioned medium harvested by rat C6 glioma cell cultures as well as the molecular pathways that direct microglia toward a pro-invasive and immunosuppressive phenotype (Gabrusiewicz et al., 2011; Ellert-Miklaszewska et al., 2013). Using co-cultures of the human microglia cell line CHME-5 and the rat glioma C6 cells, Bouzier-Sore and co-workers showed that microglial activation after exposure to C6 cells is bi-phasic, with a prior transitory phagocytic activated phenotype (M2) followed by a reversal to a non-phagocytic status (M1) (Voisin et al., 2010). Microglia express a variety of receptors on their surface, which can be activated by signals from glioma cells (Okada et al., 2009; Held-Feindt et al., 2010). For example glioma cells produce cytokines, such as IL-10, IL-4, IL-6, TGFβ and prostaglandins E2 (Rolle et al., 2012). These factors are able to activate microglial cells; in particular, IL-4 causes alternative activation (M2) of microglia (Gadani et al., 2012). Moreover, interactions between microglia and tumor cells are bi-directional (Galvão et al., 2013); under the influence of glioma, microglia release several classes of molecules that foster glioma growth, progression and inflammatory activation (Li and Graeber, 2012). Activated microglia can also release inflammatory products, such as nitrites, IL-10 and urea, that may exert detrimental effects on glioma cells.

A further factor of complexity comes from yet other cell types involved in multi-lateral communications. In fact malignant gliomas can cause a disruption of the BBB (blood–brain barrier), thus circulating immune cells not normally found in the CNS gain access to the tumor areas (Zhan and Lu, 2012). These include various types of T cells, macrophages and myeloid-derived suppressor cells that all together participate in the response to tumor growth (Charles et al., 2012, Kushchayev et al., 2012). These cells tend to secrete anti-inflammatory or pro-inflammatory cytokines, peptides and proteins that can directly activate glioma cells. Additionally, the glioma is invaded or surrounded by reactive astrocytes, which contribute to the inflammatory milieu as well (Rivera-Zengotita and Yachnis, 2012; O’Brien et al., 2013).

In the present paper, we have compared the effects of different conditioned medium on microglial cell biology. Conditioned medium harvested from inflammatory activated glioma cells should better mimic the situation that is expected to be found *in vivo* at the moment that a malignant glioma is diagnosed, i.e. when bi-directional communications between microglia and tumor cells are already well-established. For the first time, here we show that microglial cell activation undergoes a different fate depending on the stimulus used and, in presence of factors derived from the glioma-activated cells, they express a distinctive phenotype. The latter is substantially different from the phenotype expressed by microglial cells exposed to CM (conditioned media) collected by glioma cells under basal conditions. In particular, both CM-induced morphological changes in microglial cells, and increased cell viability and proliferation. However, while C-CM (control-conditioned media) tend to induce an M2b polarization of microglia, with production of free oxygen radicals and a significant up-regulation of IL-10 gene expression, CM obtained after glioma stimulation with LI-CM [LPS (lipopolysaccharide)–IFNγ (interferon γ)-conditioned media] induced a significant up-regulation of iNOS (inducible nitric oxide synthase), ARG (arginase) and IL-10 gene expression, indicating the induction of a mixed polarization profile, i.e. both M1 and M2a/b (mixture of polarization phenotypes) phenotypes.

## MATERIALS AND METHODS

### Materials

Cell culture reagents [DMEM (Dulbecco's modified Eagle's medium), DMEM-F12 and FBS] were from Invitrogen Corporation. Antibiotics were from Biochrom AG. Bacterial endotoxin LPS (*Salmonella typhimurium*) was from Sigma-Aldrich. The rat recombinant IFNγ was purchased from Endogen (Pierce Biotechnology). β-actin (clone AC-74) mouse monoclonal antibody was from Sigma Aldrich; rabbit polyclonal anti-phospho [Ser^2448^] mTOR (mammalian target of rapamycin) was purchased from Novus Biological; mouse monoclonal anti-human CD68 was purchased from Dako Italia.

### Cell cultures

#### Microglia

Primary enriched cultures of rat glial cells, microglia and astrocytes, were prepared as previously described (Dello Russo et al., 2004). In particular, microglial cells were obtained by mixed cultures of cortical glial cells (at *in vitro* day 14), by gentle shaking. Cells were plated in 96-well plates at a density of 3×10^5^ cells/cm^2^ using 100 μl/well DMEM-F12, containing 10% (v/v) FBS and antibiotics. Under these conditions, the cultures were 95–98% CD11b positive.

#### C6 glioma cells

**Figure 1:**
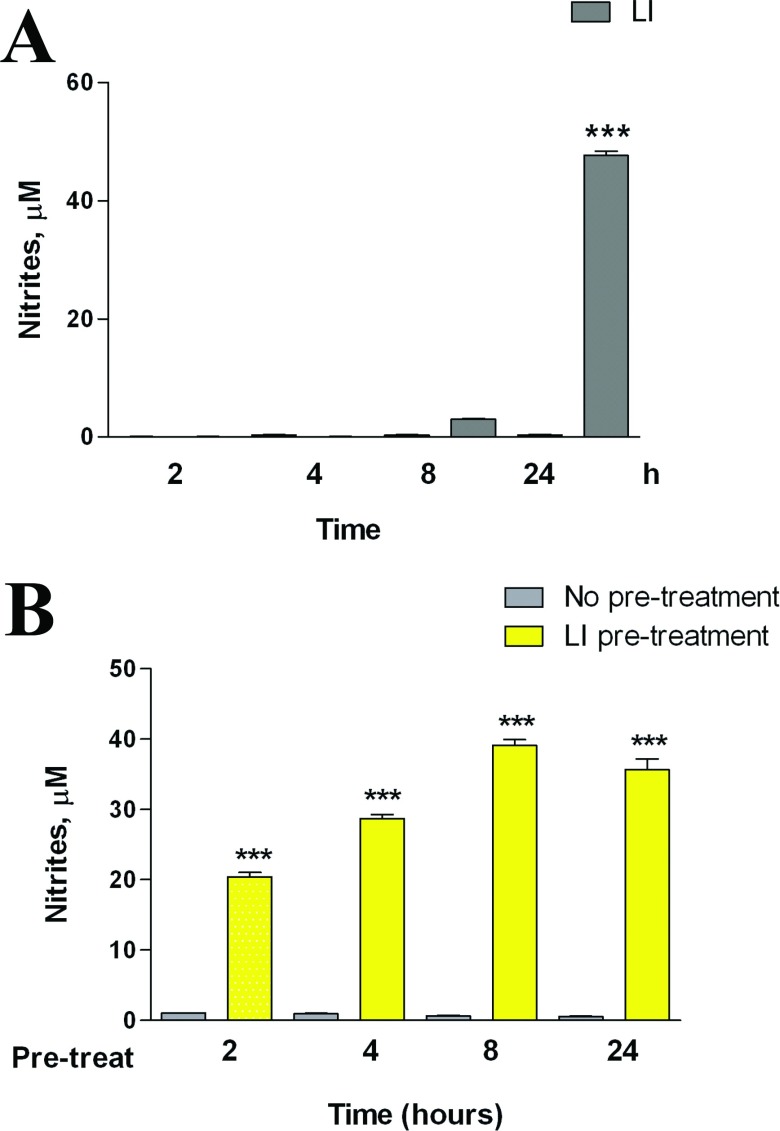
Effects of LI on C6 NO production (**A**) C6 glioma cells were activated for different times, as indicated, in presence of LI. (**B**) C6 glioma cells were pre-incubated for different times, as indicated in presence of LI and at the end of the pre-incubation time, cells were washed three times with PBS and the medium was replaced by fresh plain medium for the subsequent 24 h. NO production was assessed indirectly by measurement of nitrites (the Griess method). Data are expressed as means±S.E.M. (*n*=6). Results were analyzed by two-way ANOVA followed by the Bonferroni's *post hoc* test. ***P<0.001 versus control.

C6 glioma cells were passed once a week and were prepared as previously described (Lisi et al., 2011). CM from the activated C6 glioma cells was generated following a protocol aimed to remove the proinflammatory stimulus from the medium. Briefly, in preliminary experiment microglia cells were incubated in the presence of 1 μg/ml LPS and 10 UI/ml IFNγ for various times (Figure 1A), then this medium was removed and replaced with complete culture medium. After a 24-h conditioning period, this medium was collected and used in part to assess NO release (Figure 1B), and in part was stored at −80°C until the experiments on microglia were performed.

Based on the results shown in Figure 1, the C6-CM were prepared as it follows:

A) C-CM: 4 h incubation in the plain medium, followed by three washes with PBS (phosphate-buffered saline) and addition of fresh plain medium for 24 h. After this second period of incubation, the CM was collected, centrifuged to remove cellular debris and stored as C-CM.

B) LI-CM: 4 h incubation with LI, followed by three washes with PBS and addition of fresh plain medium for 24 h. After this second period of incubation, the CM was collected, centrifuged and stored as LI-CM.

Both CM were stored at −80°C until the experiments on microglial cells were performed.

### DCF (2′,7′-dichlorofluorescein) assay

Reactive free radicals were measured using H_2_DCF-DA [2′,7-dichlorodihydrofluorescein diacetate (Invitrogen)]. At the end of the experiment, the incubation medium was replaced by BSS {balanced salt solution [124 mM NaCl, 5.8 mM KCl, 10 mM dextrose, 20 mM Hepes, 0.3 mM CaCl_2_(H_2_O)_2_]} for 30 min. At the end of this pre-incubation period, 20 μM H_2_DCF-DA was added to the cells, which were incubated for additional 45 min at 37°C. The fluorescence signal due to H_2_DCF-DA oxidation within the cells was quantified using a spectrophotometric microplate fluorescence reader (PerkinElmer Inc.), using 485 nm as excitation and 535 nm as emission wavelength.

### Nitrite assay

iNOS activity was assessed indirectly by measuring nitrite accumulation in the incubation media. Briefly, an aliquot of the cell culture media (80 μl) was mixed with 40 μl Griess Reagent (Sigma-Aldrich) and the absorbance measured at 550 nm in a spectrophotometric microplate reader (PerkinElmer Inc.). A standard curve was generated during each assay in the range of concentrations 0–100 μM using NaNO_2_ (Sigma-Aldrich) as standard. In this range, the standard detection resulted linear and the minimum detectable concentration of NaNO_2_ was ≥3.12 μM. In the absence of stimuli, basal levels of nitrites were below the detection limit of the assay at all the time points studied.

### mRNA analysis in real-time PCR

Total cytoplasmic RNA was extracted using the RNeasy Micro kit (Qiagen), which included 15 min DNAse treatment. RNA concentration was measured using the Quant-iT™ RiboGreen® RNA Assay Kit (Invitrogen Corporation). A standard curve in the range of 0–100 ng was run in each assay using 16S and 23S rRNA (ribosomal RNA) from *Escherichia coli* as standard and provided by the kit. Aliquots (0.15 μg) of RNA were converted to cDNA using random hexamer primers. Quantitative changes in mRNA levels were estimated by real-time PCR (Q-PCR) using the following cycling conditions: 35 cycles of denaturation at 95°C for 20 s; annealing and extension at 60°C for 20 s; using the Brilliant III Ultra-Fast SYBR® Green QPCR Master Mix (Stratagene). PCR reactions were carried out in a 20 μl reaction volume in a MX3000P real-time PCR machine (Stratagene). Primers used for the evaluation of gene expression are reported in Table 1. Relative mRNA concentrations were calculated from the take-off point of reactions (threshold cycle, Ct) using the comparative quantitation method performed by Stratagene software and based upon the −ΔΔCt method. This analysis approximates a given sample's target mRNA (e.g., iNOS) level relative to the mean of the target mRNA levels in untreated controls (‘calibrator’ value), thus permitting statistical analysis of deviation from the mean even among the controls. Ct values for α-tubulin expression served as a normalizing signal. In each assay, the PCR efficiency was also calculated using serial dilution of one experimental sample; efficiency values between 94 and 98% were found for each primer set and taken into account for the comparative quantitation analysis (Dello Russo et al., 2009).

**Table 1:** Primer sets used for Q-PCR analysis.

Genes	Forward primers	Reverse primers	Product length (base pair)
α-TUB	CCC TCG CCA TGG TAA ATA CAT	ACT GGA TGG TAC GCT TGG TCT	110
iNOS	CTG CAT GGA ACA GTA TAA GGC AAA C	CAG ACA GTT TCT GGT CGA TGT CAT GA	230
IL-10	CAG CTG CGA CGC TGT CAT CGA	GCA GTC CAG TAG ATG CCG GGT G	198
ARG1	TGC CCT CTG TCT TTT AGG GC	CCT CGA GGC TGT CCC TTA GA	165
COX1	CCT CAC CAG TCA ATC CCT GT	AGG TGG CAT TCA CAA ACT CC	231
COX2	GCA TTC TTT GCC CAG CAC TTC ACT	TTT AAG TCC ACT CCA TGG CCC AGT	98
CD86	CCA GAT TGC AGG TCC CAG TT	TCG ACT CGT CAA CAC CAC TG	152
TGF-β	CATG GAG CTG GTG AAA CGG A	CGG GTG ACT TCT TTG GCG TA	219
IL-1β	AGG CTG ACA GAC CCC AAA AGAT	CTC CAC GGG CAA GAC ATA GGTA	157
TNF-α	CCA CCA AGC GGA GGA GCA GC	TCG GCT GAC GGT GTG GGT GA	187

### Cell viability measurement

Microglial viability was assessed by reduction of the tetrazolium compound MTS [3-(4,5-dimethylthiazol-2-yl)-5-(3-carboxymethoxyphenyl)-2-(4-sulfophenyl)-2*H*-tetrazolium, inner salt] contained in the CellTiter AQueous One Solution Reagent (Promega). For this assay, cells were seeded in 96-well plates. At the end of the experimental procedure, 20 μl of MTS reagent were added to the cells that were further incubated for 2 h. Living cells bio-reduces yellow MTS into a purple soluble formazan product with an absorbance peak at 492 nm, that was read in a spectrophotometric microplate reader (PerkinElmer Inc.)

### Cell proliferation assay

Microglial proliferation rate was determined using a non-radioactive proliferation assay (Exalpha Biological Inc.), used according to the manufacturer's instructions. This ELISA assay measures the incorporation of BrdU (bromodeoxyuridine or 5-bromo-2-deoxyuridine) into newly synthesized DNA of actively proliferating cells. Microglial cells were incubated in plain medium or C-CM, LI-CM for 48 h. BrdU solution was added 32 h later directly in the incubation medium, and cells were kept in the incubator for the remaining 16 h. At the end of the incubation time, the medium was removed and cells were fixed. BrdU immunoreactivity was measured using a specific primary antibody provided by the kit. Data are expressed as percentage of control values.

### Immunocytochemistry

For immunocytochemistry, microglia was plated at a density of 3.5×10^5^ cells/well on the coated glass coverslips and treated as indicated for 48 h. At this time, cultures were washed with PBS containing Ca^2+^ and Mg^2+^ (PBS-w) and fixed in 4% (w/v) paraformaldehyde for 15 min. After fixation, cultures were washed three times with PBS-w and permeabilized with 0.1% Triton-X-100 for 5 min; then washed and blocked with 1% (w/v) BSA in PBS-w for 30 min. Phalloidin-TRITC (1 μM) in PBS-w containing 0.1% BSA and 0.1% (v/v) Triton X-100 was added for 2 h. After incubation, DAPI (4′,6-diamidino-2-phenylindole) was added for 10 min and then washed twice with PBS-w. Coverslips were mounted using Vectashield mounting media. Images were obtained on a Nikon Eclipse TE300 inverted fluorescence microscope equipped with a Cool SNAP professional digital camera and LUCIA-G/F imaging software.

### Western immunoblot

The cells were lysed in RIPA buffer [1 mM EDTA, 150 mM NaCl, 1% (v/v) igepal, 0.1% (w/v), SDS, 0.5% (w/v) sodium deoxycholate, 50 mM Tris–HCl, pH 8.0] (Sigma-Aldrich) containing protease inhibitor cocktail diluted 1:250 (Sigma-Aldrich). The protein content in each sample was determined by Bradford's method (Biorad) using bovine serum albumin as standard. A 10-μg aliquot of protein was mixed 1:2 with 2X Laemmli Buffer (Biorad), boiled for 5 min, and separated through SDS/10% PAGE gels. Apparent molecular weights were estimated by comparison with colored molecular weight markers (Sigma Aldrich). After electrophoresis, proteins were transferred to polyvinylidene difluoride membranes by semi-dry electrophoretic transfer (Biorad). The membranes were blocked with 10% (w/v) low-fat dried skimmed milk powder in TBST (10 mM Tris, 150 mM NaCl, 0.1% (v/v) Tween-20, pH 7.6) (Biorad) for 1 h at room temperature (25°C), and incubated in the presence of the primary antibody overnight with gentle shaking at 4°C. Primary antibodies for phosphorylated-mTOR, β-actin (Sigma-Aldrich) were used at the final concentration of 1:1000. Primary antibodies were removed, membranes washed three times in TBST, and further incubated for 1 h at room temperature in the presence of specific secondary antibody, anti-rabbit for mTOR and anti-mouse for β-actin IgG-HRP (horseradish peroxidase) conjugated (Sigma-Aldrich), diluted 1:15,000. Following three washes in TBST, bands were visualized by incubation in ECL reagents (GE Healthcare) and exposure to Hyperfilm ECL (GE Healthcare). The same membranes were washed three times in TBST, blocked with 10% (w/v) low-fat dried skimmed milk powder in TBST for 1 h at room temperature and used for β-actin immunoblot.

### Tissue preparation and immunohistochemistry

Human tumor tissue obtained from surgical resection of patients with IV grade glioblastoma were fixed in 4% paraformaldehyde in 0.1 M phosphate buffer pH 7.6 overnight at 4°C. Dehydration of tissue was through a series of 80%, 95% (v/v) ethanol 1 h each followed by 100% ethanol overnight. Two 100% (v/v) xylene washes were done for 1 h each and then 1 h in 60°C Paraplast Plus (Tyco/Healthcare). After a change of Paraplast Plus, tissue was incubated in a 60°C vacuum oven for 2 h prior to placing in molds to cool and solidify. Sections, 5-μm thick, were cut and mounted. Sections were deparaffinized by drying on superfrost plus slides (Fisher), heating at 56°C overnight, and then washing through mixed xylenes, 100% ethanol, 95% ethanol, ddHO. Slides were immersed in 10 mm citrate buffer, pH 6.0, dry heated for 10 min each to unmask antigen sites, and then cooled and washed in PBS. Endogenous peroxidase activity was inhibited by rinsing the slides in 3% hydrogen peroxide for 5 min. Non-specific binding was blocked by 5 min incubation with the Super Block Solution (ScyTek Laboratories). After washing in PBS, sections were incubated for 30 min at room temperature with monoclonal Mouse Anti-Human CD68 (1:100; Dako Italia). Sections were washed extensively with PBS and subsequently treated with the Ultra Tek Anti-Polyvalent kit (ScyTek Laboratories). Finally, sections were treated with 3,3′-diaminobenzidine as chromogen, contrasted with hematoxylin–eosin and mounted.

### Data analysis

All experiments were performed using five to six replicates per each experimental group, and repeated at least three times. For the RNA analysis, the samples were assayed in triplicates, and the experiments were repeated at least twice. Data were analyzed by one- or two-way ANOVA followed by the Bonferroni's *post hoc* test or by unpaired *t* test where appropriate. *P* values<0.05 were considered significant.

## RESULTS

As shown in Figure 1, C6 cells can be activated in response to a standard pro-inflammatory stimulus, namely LI (LPS at 1 μg/ml plus IFNγ at 10 units/ml). In particular, we evaluated the activity of the iNOS, which was significantly increased after an incubation period of 24 h (Figure 1A). The effects of LI were long-lasting; in fact, pre-exposure of C6 cells to the pro-inflammatory stimulus increased the levels of nitrites in the following 24 h incubation in the plain medium, regardless of the length of pre-incubation (Figure 1B). Based on these results, we prepared glioma CM, pre-incubating for 4 h the C6 cells with LI (or plain medium, in order to assess the effect of glioma under basal condition). After the pre-incubation time, cells were carefully washed and incubated for additional 24 h to generate either LI-CM (CM harvested LI-activated glioma cells) or C-CM (CM collected from glioma cells kept under basal conditions). To test the effect of glioma soluble factors on microglial cells, 50% of CM (either C-CM or LI-CM) mixed to 50% microglia incubation medium [DMEM-F12 with 10% (v/v) FBS] was used; whereas a medium consisting of 50% C6 cell incubation medium (i.e. DMEM with 1% FBS) and 50% DMEM-F12 (10% FBS) was prepared for controls.

**Figure 2:**
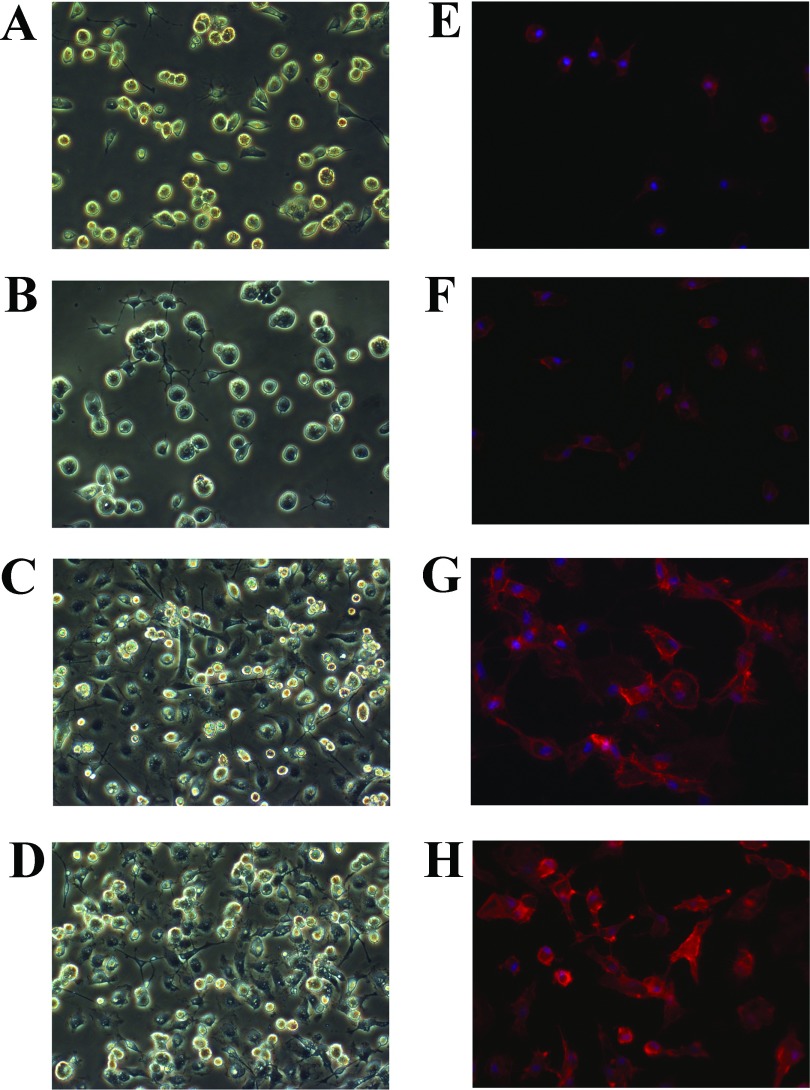
Morphology and F-actin immunostaining of primary cultures of microglia cells (**A**–**D**) Morphology of a primary culture of rat microglia cells, observed by phase-contrast microscopy; and (**E**–**H**) morphological modifications of microglial cultures, observed 24 h post-treatments with a staining of F-actin with phalloidin-TRIC and fluorescence microscopy. (**A**,**B**, and **E**,**F**) Cells under basal conditions or after stimulation with (**C** and **G**) C-CM or (**D** and **H**) LI-CM for 24 h.

**Figure 3:**
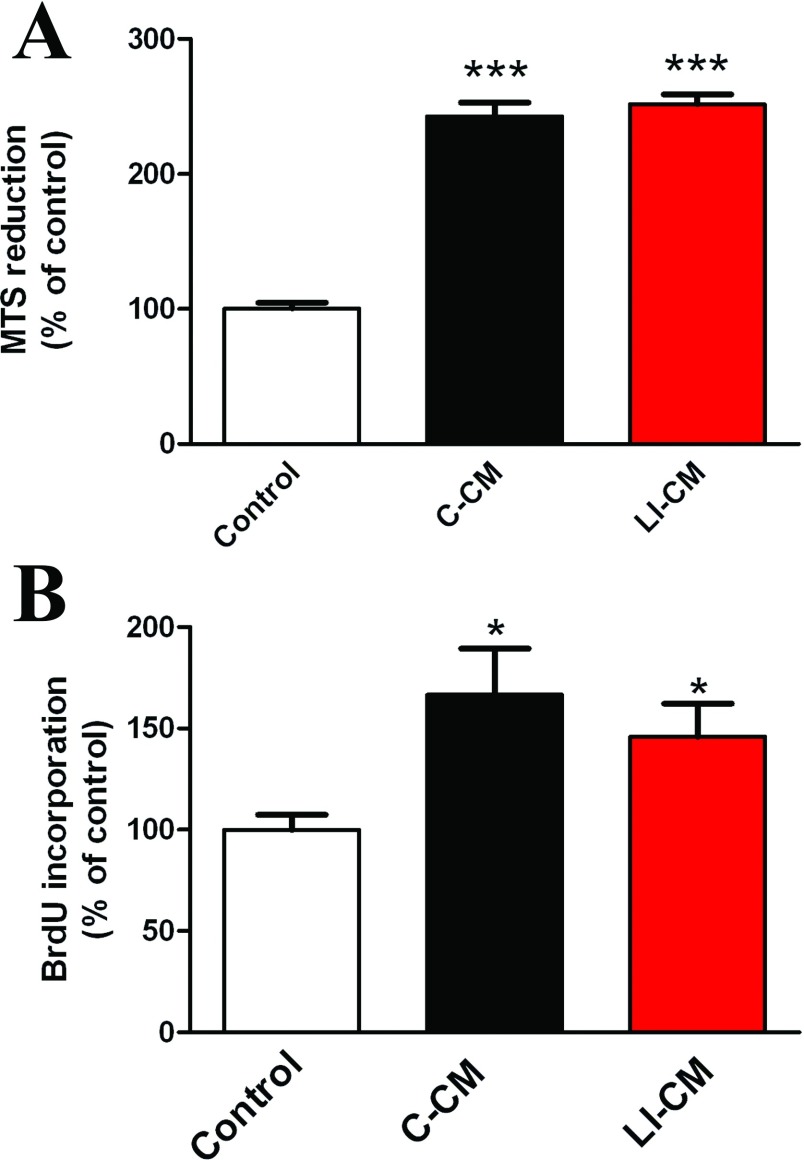
Effects of C6 CM on microglial viability and proliferation Microglia cells were treated for 48 h with C-CM or LI-CM. Effects on cell viability were assessed by the MTS reduction assay, whereas the effects on cell proliferation were assessed by BrDU incorporation. Data were analyzed by one-way ANOVA followed by the Bonferroni's *post hoc* test. ***P<0.001,*P<0.05 versus Control.

First, we tested the effects of C-CM and LI-CM on microglial morphology, cell viability and proliferation. Microglial cultures exposed for 24 h to 50% C-CM or 50% LI-CM did undergo morphological transformation into amoeboid cells, as shown by phase contrast microscopy (Figure 2) and F-actin staining with phalloidin-TRIC (Figure 2). The switching of incubation medium from 100% DMEM-F12 (10% FBS) to 50% DMEM-F12 (10% FBS)/ 50% DMEM (1% FBS) for 24 h had no effect on microglia morphology (Figures 2B–2F); thus supporting the use of this medium as control treatment in subsequent experiments. As expected, a 48-h exposure of microglial cells to CM harvested from both resting and LI-activated C6 cells increased microglial viability (Figure 3A) and cell proliferation (Figure 3B). No significant differences were observed between the two CM on these two parameters.

**Figure 4:**
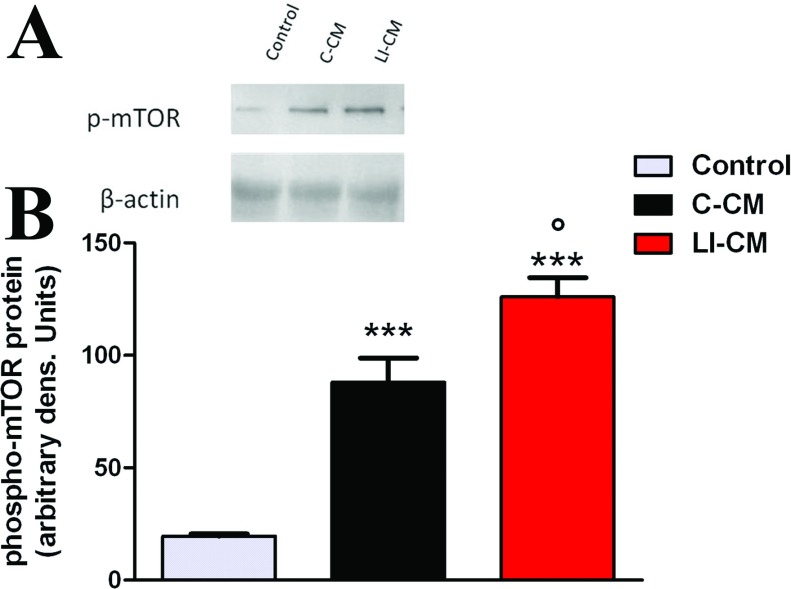
Analysis of mTOR phosphorylation during microglial activation (**A**) Whole-cell lysates were prepared from microglial cells incubated for 2 h as indicated. Equal amounts of proteins were analyzed by western blot for phosphorylated mTOR kinase (p-mTOR), upper gel. The same blots were subsequently probed for β-actin, lower gel. (**B**) Quantitation of densitometry where p-mTOR values are reflected relative to those for β-actin. Data are expressed as means±S.E.M. of *n*=1 replicate for each group, each assayed in triplicates. Representative of two different experiments. Data were analyzed by one-way ANOVA followed by the Bonferroni's *post hoc* test. ***P<0.001 versus Control, °*P*<0.05 versus C-CM.

We have recently shown that the mTOR kinase is important to maintain microglial viability and cell proliferation; moreover, it is involved in cytokine-dependent microglial pro-inflammatory activation (Dello Russo et al., 2009). In light of this evidence, the effect of CM on the activation status of mTOR was assessed. As shown in Figure 4, we found a significant difference among the two CM in the magnitude of phosphorilation of mTOR kinase at Ser^2448^, which is an index of mTOR activation: in 2-h experiments, we detected a 4-fold and a 6-fold increase in mTOR activation after the exposure of microglia to C-CM and LI-CM, respectively, compared with the control medium. Thus, although responses induced by exposure to CMs are qualitatively similar, LI-CM elicited a net +50% increase in mTOR activation compared with C-CM. A not-conditioned medium containing 1 ng/ml LPS, used as a positive control, induced a 1.5-fold increase in the activation of mTOR, whereas the exposure to a medium containing 50% DMEM (1% FBS) reduced mTOR activation by roughly 50% (results not shown).

**Figure 5:**
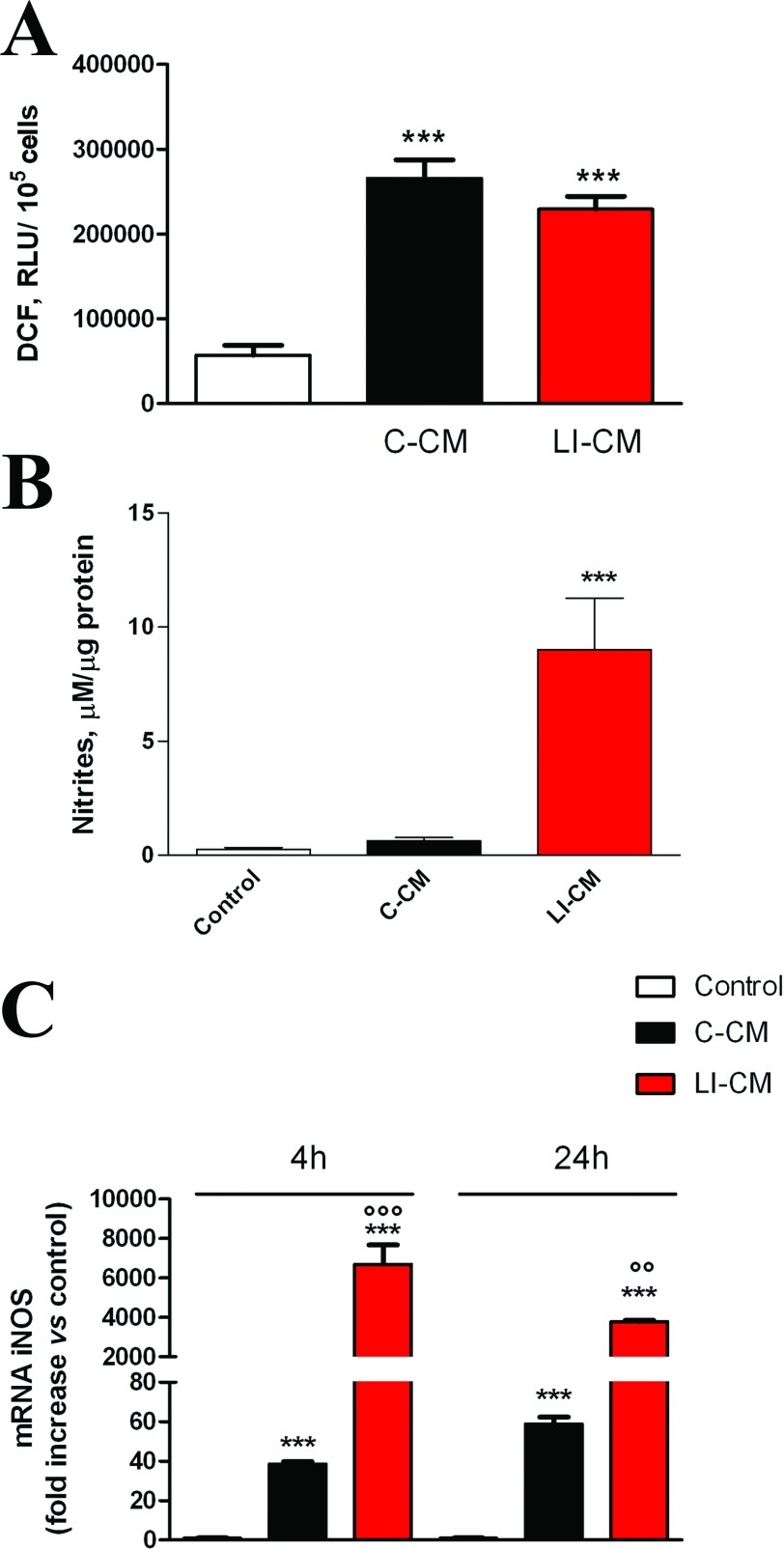
Effects of the CM on iNOS expression and activity (**A**) Reactive free radicals were assessed by the oxidation of H_2_DCF added to the cells at the end of the experiment. (**B**) Cells were stimulated with C-CM or LI-CM as indicated. After 48 h incubation, the medium was used for nitrite assessment whereas cells were lysed in 200 mM NaOH and protein content was evaluated by the Bradford's method. Results are expressed as μM of nitrites/μg of proteins; data are means±S.E.M. (*n*=6). ***P<0.001 versus control. (**C**) Total cytosolic RNA was prepared from control, or microglial cells treated with C-CM or LI-CM for different times, and used for real-time (Q)-PCR analysis of NOS2 expression. Data are expressed as fold change versus control, taken as calibrator for comparative quantitation analysis of mRNA levels. Each sample was measured in triplicate, the experiment was repeated two times with similar results. Data are means±S.E.M., and were analyzed by two-way ANOVA followed by the Bonferroni's *post hoc* test. ***P<0.001 versus control and °°°*P*<0.001, °°*P*<0.01, versus C-CM.

**Figure 6:**
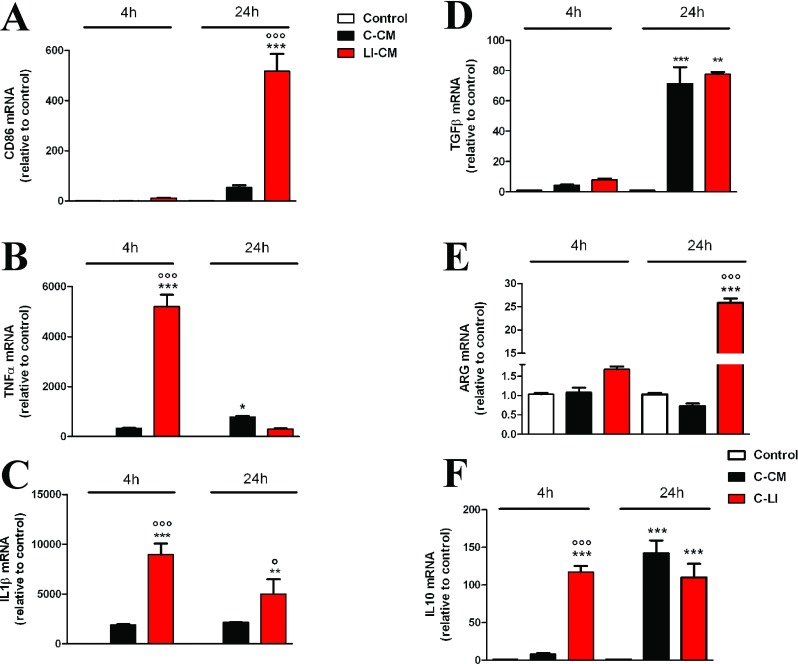
Effects of the CM on inflammatory gene expression Total cytosolic RNA was prepared from the control, or microglial cells treated with C-CM or LI-CM for different times, and used for real-time Q-PCR analysis of different inflammatory genes, (**A**) CD86, (**B**) TNFα, (**C**) IL-1β, (**D**) TGFβ, (**E**) arginase I,ARG and (**F**) IL-10. Data are expressed as fold change versus control, taken as calibrator for comparative quantitation analysis of mRNA levels. Each sample was measured in triplicate, the experiment was repeated two times with similar results. Data are means±S.E.M., and were analyzed by two-way ANOVA followed by the Bonferroni's *post hoc* test. ***, *P*<0.001, **P<0.01, *P<0.05 versus control; °°°*P*< 0.001, °*P*<0.05 versus C-CM.

A subsequent set of experiments was carried out to assess the effect of glioma CM on microglial pro-inflammatory activity. First, we measured total levels of reactive species, both nitrogen and oxygen-free radicals, using the fluorescent probe H_2_DCF-DA (LeBel et al., 1992). Both CMs were able to increase total ROS (reactive oxygen species) levels (Figure 5A). However, the exposure of microglial cells to C-CM significantly increased the expression of iNOS (Figure 5C) with no sizable effect on iNOS activity (Figure 5B), whereas on the contrary LI-CM was found able to increase both iNOS expression and enzyme activity (Figures 5B and 5C). Furthermore, LI-CM significantly increased the expression of different genes involved in both classic (M1) and alternative (M2) activation of microglial cells. In response to LI-CM, we detected a significant up-regulation of the following genes: CD86, an M1/M2 polarization marker (Figure 6A); TNF-α (Figure 6B) and IL-1β (Figure 6C), markers of M1 classic activation; TGFβ (Figure 6D), an M2 marker; ARG (Figure 6E), an M2a polarization marker; and IL-10 (Figure 6F) a marker of the M2b activation status. Conversely, C-CM mostly induced a significant up-regulation of genes involved in the induction of the alternative M2b activation phenotype, such as IL-10 (Figure 6F) and TGFβ (Figure 6D). Finally, C-CM had no stimulatory effect on ARG expression, whereas a 20% reduction was observed after 24 h (Figure 6E). Taken together, these data show that soluble factors from glioma cells promote microglial inflammatory activation, even though C-CM seems to specifically induce M2b-type activation, while LI-CM promote a mixed phenotype, with M1 and M2a/2b polarized cells.

**Figure 7:**
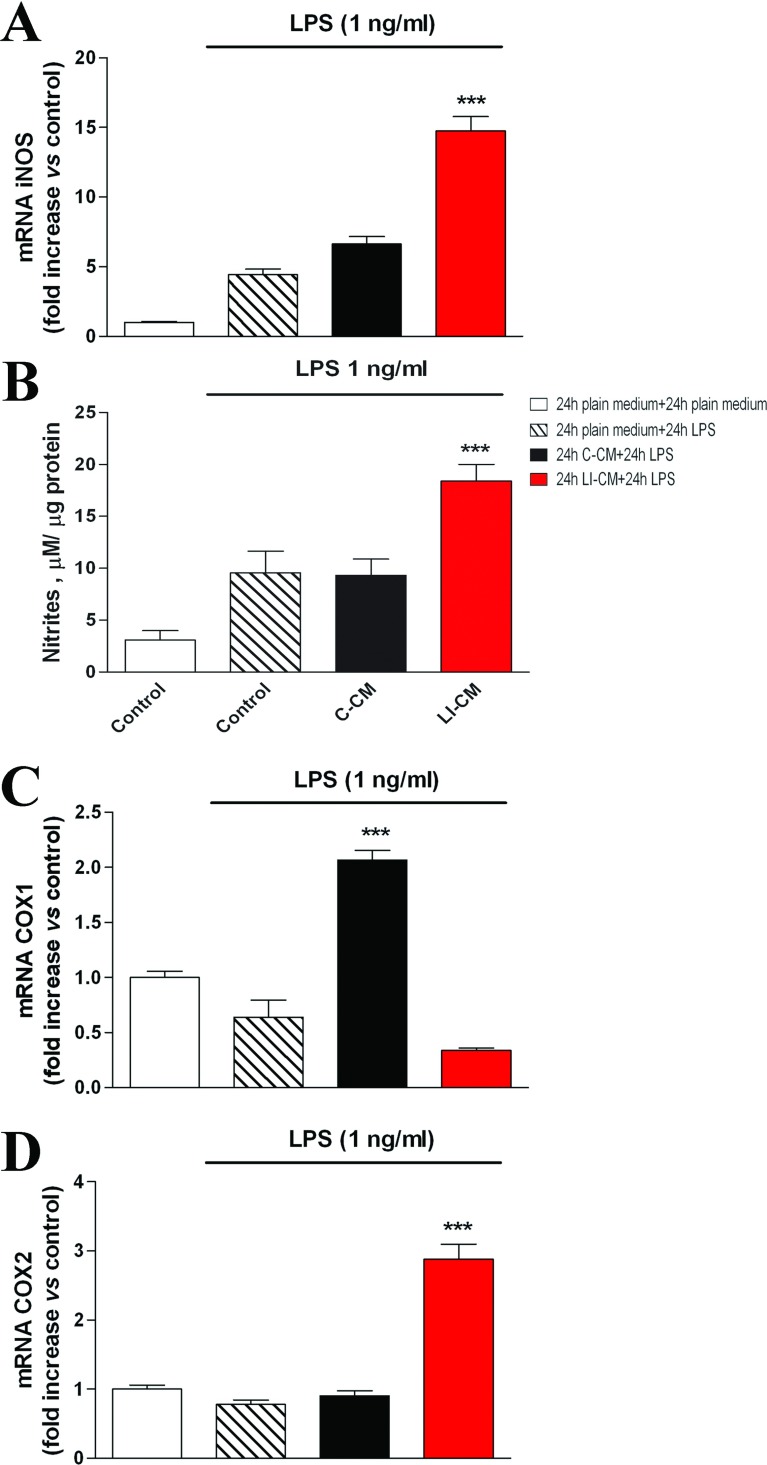
Effects of LPS on iNOS and COX in microglia pre-treated with C6 CM Total cytosolic RNA was prepared from Control, or microglial cells pre-treated with C-CM or LI-CM for 24 h and subsequently stimulated with LPS for additional 24 h. The RNA was used for Q-PCR analysis of iNOS (**A**), COX1 (**B**) and COX2 (**C**) gene expression. Data are expressed as fold change versus control cells (white column, microglial cells not exposed neither to C6-CM nor to LPS), taken as calibrator for the comparative quantitation analysis of mRNA levels. Each sample was measured in triplicate, the experiment was repeated two times with similar results. Data are means±S.E.M. ***P<0.001 versus control; one-way ANOVA followed by the Bonferroni's *post hoc* test. (**B**) Cells were stimulated with C-CM or LI-CM for 24 h as indicated and then restimulated with LPS for 24 h. At the end of this second incubation time, the medium was used for nitrite assessment, whereas cells were lysed in 200 mM NaOH and protein content evaluated by the Bradford's method. Results are expressed as μM of nitrites/μg of proteins; data are means±S.E.M. (*n*=6). ***P<0.001 versus control.

Complex glioma-microglia interactions are also confirmed by the finding that pre-exposure of microglial cells to C6 CMs induces different responses to a classic M1 pro-inflammatory stimulus, as LPS. As shown in Figure 7, a 24-h challenge with LPS did not modify iNOS expression (A) and activity (B) in microglia pre-treated for 24 h with C-CM. Conversely, a 24-h pretreatment with LI-CM was associated to a 3-fold increase in iNOS expression, and a 2-fold increase in the amount of nitrites, induced by LPS with respect to controls (Figure 7). In a further series of experiments, we investigated the effects of C6-CMs pretreatment on COX (cyclooxygenase) isoforms gene expression. Pretreatment of microglial cells with C-CM results in a selective up-regulation of constitutive COX (COX1), but not COX-2, in response to LPS (Figure 7C), whereas on the contrary pre-incubation with LI-CM led to the up-regulation of pro-inflammatory COX2, but not COX1 (Figure 7D).

**Figure 8:**
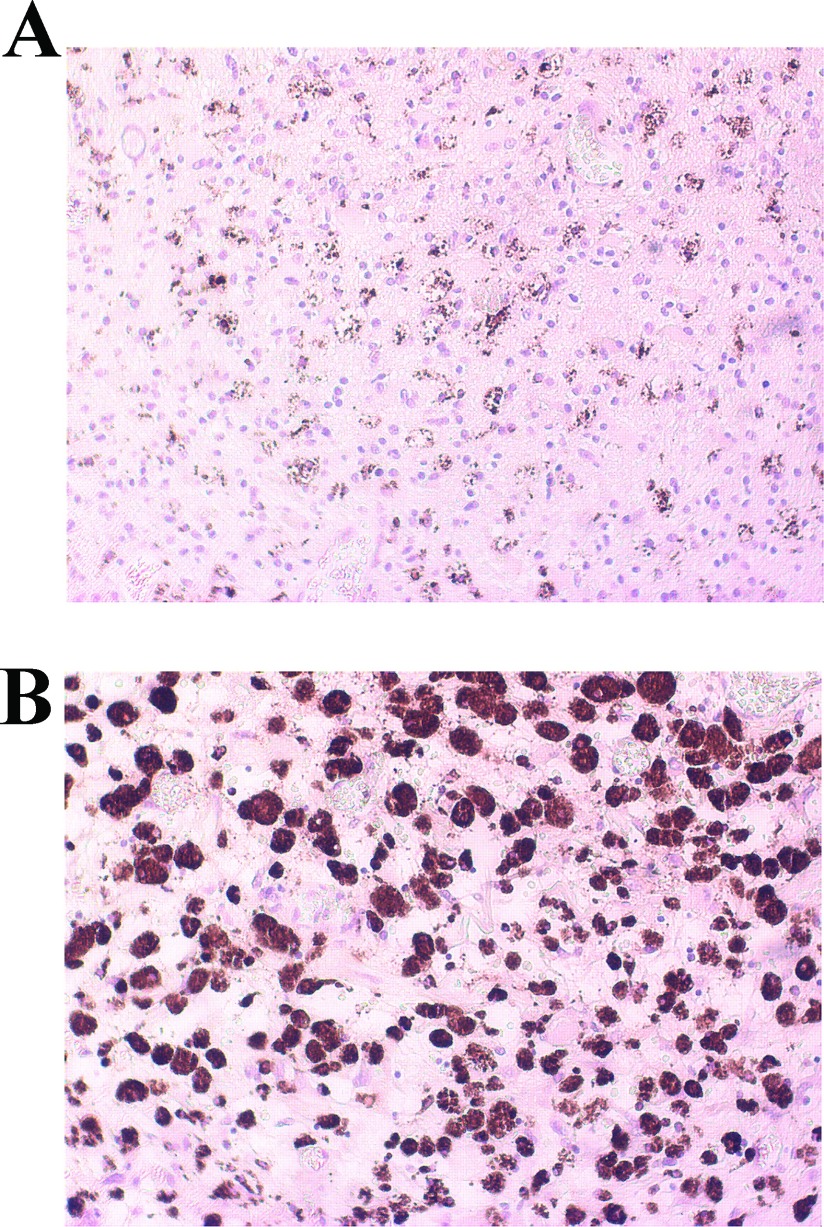
Microglia cells in human glioblastoma tissue Human tumor sections from four different patients were stained with monoclonal mouse anti-human CD68 [PG-M1 clone] to observe the presence of phagocytic microglia/macrophage. Albeit in different amount, CD68 positive cells were found in all stained samples. Representative sections are shown 20× (**A**) and 40× (**B**).

Finally, sections of glioma tissue obtained from surgical resection of patients diagnosed with IV grade glioblastoma (GBM) were stained for CD68 (PG-M1 clone), a marker of phagocytic activity of macrophages/microglia (Boche et al., 2013). As shown in Figure 8, a diffuse infiltrate of CD68-positive cells was detected, thus supporting the hypothesis that *in vitro* findings presented here might indeed occur in the setting of human pathology.

## DISCUSSION

In the present study, we showed that media collected from both basal and pre-activated C6 glioma cells induce morphological changes in microglia cells and increase cell viability and proliferation. However, while C-CM induced a specific M2b-type polarization of microglia, characterized mainly by a significant up-regulation of IL-10 and TGFβ gene expression; LI-CM harvested from pre-stimulated (LPS–IFNγ) glioma cells led to a mixed phenotype, M1 and M2a/M2b, as indicated by the concurrent up-regulation of iNOS, TNFα, IL-1β, TGFβ, ARG, IL-10 and the M1/M2 polarization marker, CD86. Incubation medium used for C6 cell cultures (DMEM with 1% FBS) differs from that used for microglia (DMEM-F12 with 10% FBS); being the most important difference between them the percentage of serum added to the medium, which can directly affect cell viability as well as the response to pro-inflammatory stimuli. Therefore one might expect that the switch of the microglial cultures to a medium containing 50% of C6 cell incubation medium could *per se* induce morphological changes. However, this is not the case since no significant differences were associated to the different type of medium (Figures 2A, 2B, 2E and 2F). Therefore the 50% C6 cell incubation medium was used as control in most of the experimental procedures.

The CM from activated C6 glioma cells were generated following a more complex protocol (see the Method section), aimed to remove LPS and IFNγ from the media in order to avoid their direct effects on microglia. By using LI-CM we intended to mimic the *in vivo* condition of late-stage pathology, when gliomas cause significant breakdown of the BBB (Wolburg et al., 2012); as a consequence, circulating immune cells not normally found in the CNS invade the tumor and release factors promoting the pro-inflammatory activation of glioma cells (Zhan and Lu., 2012). Conversely, we considered the C-CM experimental paradigm as an *in vitro* correlate of early-stage glioma pathology, when glioma cells are seemingly not yet activated.

Both C-CM and LI-CM increased microglia cell proliferation in a similar manner, suggesting the similar underlying mechanisms, seemingly not involving pro-inflammatory molecular pathways. Consistent with this hypothesis, Kaminska and co-workers reported that glioma-derived soluble factors induce the activation of focal adhesion kinase, PI3K (phosphoinositide 3-kinase)/Akt (protein kinase B), ERK (extracellular-signal-regulated kinase), and P38 MAPK (mitogen-activated protein kinase) signaling pathways, resulting in enhancement of cell motility, phagocytosis, sustained proliferation and a distinct genomic response. Such activated microglial phenotype appeared to be unique, being not associated with activation of NFκB (nuclear factor κB) and Stat1 and thus up-regulation of inflammatory genes (Ellert-Miklaszewska et al., 2013).

We have recently demonstrated that mTOR kinase activation is involved in microglial cell proliferation and viability under basal conditions, as well as in pro-inflammatory activation in response to bacterial endotoxin or a mixture of inflammatory cytokines (Dello Russo et al., 2009). Phosphorylation at Ser^2448^ of the mTOR protein primarily reflects a feedback signal from the mTOR downstream target, the p70S6K (p70S6 kinase), and it is therefore considered a reliable marker of mTOR activation within the cells (Rosner et al., 2010). Interestingly, both C-CM and LI-CM increased mTOR phosphorylation at Ser^2448^, albeit to a different extent. In this regard, we have previously observed that the effect of LPS was more marked compared with cytokines on mTOR phosphorylation at Ser^2448^, suggesting that different stimuli can induce a different level of mTOR phosphorylation, possibly because of different signaling mechanisms. Moreover, we can speculate that different magnitudes of mTOR phosphorylation can be associated to different pattern of microglial activation. In support of this hypothesis, we observed that the higher level of mTOR activation induced by LI-CM in comparison with C-CM (or else to the direct effect of 1 ng/ml LPS) resulted in the activation of a larger array of inflammatory mediators. In particular, the exposure of microglial cells to C-CM, despite a modest albeit significant increase in iNOS expression (Figure 5C), did not modify iNOS activity (Figure 5B), whereas it significantly increased IL-10 and TGFβ gene expression after 24 h (Figures 6F and 6D). Conversely, LI-CM was found to increase both iNOS expression and the enzyme activity (Figures 5B and 5C), and was also able to increase other markers of M1 inflammatory activation, such as TNFα and IL-1β (Figures 6B and 6C). However, LI-CM also significantly increased the expression of ARG, IL-10 and TGFβ (Figures 6D–6F) as well as the expression of the M1/M2 marker CD86 (Figure 6A). In this regard, Chhor et al. (2013) have previously shown that iNOS is a marker of M1 activation, ARG of M2a activation and IL-10 of M2b activation. Therefore, our results, based on this classification, suggest that C-CM is able to polarize the microglia cells mainly versus the M2b alternative status of activation, whereas LI-CM induces a switch toward M1 and M2a phenotypes. Interestingly, Chhor et al. (2013) also showed that a pre-exposure of microglial cells to the M2a-inducing stimulus IL-4 before the LPS challenge failed to prevent the typical induction of M1 markers (COX2, iNOS). Similarly, in our model the administration of LPS after LI-CM (i.e. an M2a-inducing condition) significantly increased COX2 gene expression and both iNOS gene expression and activity. On the other hand, a previous exposure to C-CM, taken as an M2b-inducing condition, prevented the typical effects of LPS, i.e. microglial expression of M1 markers. Similar findings were also obtained by Hu et al. (2012) in focal cerebral ischemia. Consistent with our data, Voisin et al. (2010) found that 45% of the microglia appeared to be in a low phagocytic state (M1) and 55% of the cells had a high capacity for phagocytosis (M2) at the beginning of exposure to both isolated culture conditions and microglia-glioma co-cultures (human microglial cell line, CHME5 and rat glioma C6 cells). During the first 3 h of co-culture, the population in a low phagocytic state tended to decrease, whereas the opposite occurred with the population in a higher phagocytic state; however, after 6 h of co-culture, this trend tended to reverse so that, after 24 h of co-culture, a large increase in the M1 population and a drastic reduction of the M2 phagocytic one were observed.

At a late stage of this research we thought it was interesting to seek for any parameter of human pathology that could possibly correlate with our *in vitro* findings on the animal model. Interestingly, in human glioma tissue obtained from surgical resection of patients with IV grade glioblastoma, we detected a significant amount of CD68 positive cells. The latter is a marker of macrophage/microglial phagocytic activity, thus a not specific index of M1/M2 activated cells. The presence of CD68 positive cells in all the analyzed tumor samples, albeit in different amount, suggest the relevance of activated microglia/macrophages in the human pathology as well. However, further characterization is necessary in order to correlate the *in vitro* data to the human pathology with respect to the extent of microglial polarization at different stages of disease and/or the possible neoplastic nature of CD68 positive cells, as hypothesized by the group of Seyfried (Huysentruyt et al., 2011).

In conclusion, our data consistently show that a different degree of glioma activation can polarize microglia cells in different manners, suggesting that microglia might in turn exert different effects on glioma cells depending on the stage of disease. Additional studies are needed to fully elucidate the role of microglia within the glioma and see whether different populations differentiate over time, possibly correlating with a different outcome of the disease. It is also possible that pharmacological strategies aimed at blocking microglial differentiation and their polarization towards the M2 phenotype might have beneficial effects in the course of the cancer pathology.

## AUTHOR CONTIBUTION

Lucia Lisi, Cinzia Dello Russo and Pierluigi Navarra contributed to study concepts; Lucia Lisi and Cinzia Dello Russo helped in study design; Lucia Lisi and Egidio Stigliano assisted in data acquisition; Lucia Lisi, Cinzia Dello Russo and Libero Lauriola helped in data analysis and interpretation; Lucia Lisi and Cinzia Dello Russo assisted in statistical analysis of data; Lucia Lisi assisted in manuscript preparation; Cinzia Dello Russo assisted in manuscript editing; and Pierluigi Navarra assisted in manuscript review.
